# A highly efficient non-viral process for programming mesenchymal stem cells for gene directed enzyme prodrug cancer therapy

**DOI:** 10.1038/s41598-020-71224-2

**Published:** 2020-08-31

**Authors:** Yoon Khei Ho, Jun Yung Woo, Geraldine Xue En Tu, Lih-Wen Deng, Heng-Phon Too

**Affiliations:** Department of Biochemistry, National University of Singapore, Singapore, 119260 Singapore

**Keywords:** Gene delivery, Genetic engineering, Cancer

## Abstract

Mesenchymal stem cells (MSCs) driven gene-directed enzyme prodrug therapy has emerged as a potential strategy for cancer treatment. The tumour-nesting properties of MSCs enable these vehicles to target tumours and metastases with effective therapies. A crucial step in engineering MSCs is the delivery of genetic material with low toxicity and high efficiency. Due to the low efficiency of current transfection methods, viral vectors are used widely to modify MSCs in preclinical and clinical studies. We show, for the first time, the high transfection efficiency (> 80%) of human adipose tissue derived-MSCs (AT-MSCs) using a cost-effective and off-the-shelf Polyethylenimine, in the presence of histone deacetylase 6 inhibitor and fusogenic lipids. Notably, the phenotypes of MSCs remained unchanged post-modification. AT-MSCs engineered with a fused transgene, yeast cytosine deaminase::uracil phosphoribosyltransferase (CDy::UPRT) displayed potent cytotoxic effects against breast, glioma, gastric cancer cells in vitro. The efficiency of eliminating gastric cell lines were effective even when using 7-day post-transfected AT-MSCs, indicative of the sustained expression and function of the therapeutic gene. In addition, significant inhibition of temozolomide resistant glioma tumour growth in vivo was observed with a single dose of therapeutic MSC. This study demonstrated an efficient non-viral modification process for MSC-based prodrug therapy.

## Introduction

Currently, there are > 900 clinical trials using mesenchymal stem cells (MSCs) registered in the National Institutes of Health clinical trials database (clinicaltrials.gov)^1,2^. While MSC-based treatments are considered safe^3^, preclinical and clinical data have shown moderate effects at best and often ineffective for some indications^4,5^. It is increasingly appreciated that the therapeutic potency may be improved by using augmented MSCs preconditioned with cytokines and growth factors, abiotic conditions, pharmaceuticals and modified genetically or reprogramed^5–7^.

Due to the inherent tumour tropism, MSCs serves as an attractive cell vehicle to deliver anticancer agents specifically to tumours and their metastatic sites^8,9^. Recently, MSC-driven gene-directed enzyme prodrug therapy (GDEPT) clinical trials have presented promising results that warrant further development^10^, with other ongoing clinical trials (ClinicalTrials.gov Identifier: NCT03298763, NCT02530047, and NCT02079324). This therapeutic approach enables localized and controlled conversion of the non-toxic prodrug enzymatically in close proximity to the target cells. The ‘by-stander effect’ increases the cytotoxicity against target cells^11^. The anticancer potential of CD-producing MSCs has been demonstrated in a broad spectrum of solid cancers^11,12^, including gastric cancer^13,14^, breast cancer^15,16^, and glioblastoma^17,18^. Preclinical studies have demonstrated that cytosine deaminase/5-fluocytosine (CD/5FC) is highly robust, where as low as 4% of CD positive cells in the tumour mass is sufficient to completely eradicate the tumour^19^. A significant advancement with the CD/5FC system was the inclusion of uracil phosphoribosyl-transferase (UPRT), a pyrimidine salvage enzyme that directly converts 5FU to 5-fluorouridine monophosphate (FUMP), thus bypassing the rate-limiting enzymes dihydropyrimidine dehydrogenase and orotate phosphoribosyltransferase^20,21^. The fused transgene enhances the conversion of 5FC into its active metabolites by 30–1,500 folds in comparison to CD/5FC and 5FU^22^.

Majority of preclinical studies and clinical trials have exploited viral vectors as efficient gene delivery vehicles in modifying MSCs^10,23^. While viral gene delivery is highly efficient, there are major drawbacks which include random integration of virus vector into the host genome, which may interrupt essential gene expression and cellular processes^5,24,25^. Even with non-integrating viral vectors, safety risks of viral transduction due to possible presentation of viral antigens on transduced cells^25^ that could potentially activate an immune response in vivo following transplantation^26^. Production of viral vectors is both labour intensive and technically demanding, thus posing a challenge to scale up with increasing number of transgenes^27,28^. Furthermore, it is worthy to note that cells infected with viral vectors typically have low copy numbers (< 10 copies/cell)^29^, unlike non-viral methods where thousands of DNA copies can be delivered into individual cells, hence, increasing the payload in delivering therapeutic agents^30,31^. For these reasons, it is highly desirable to use non-viral transfection methods for MSCs^5^. Although non-viral methods may have distinct advantages in increased payload, ease of production, low cost and good safety profiles^32^, transfection of MSCs is however, generally poor in efficiency (0–35%)^33–35^.

Previously, we demonstrated a process for the efficient transfection of human bone marrow MSCs and neurons using a reporter gene. In the presence of a mixture of fusogenic lipids and a histone deacetylase inhibitor (hereby termed as Enhancer), bone marrow mesenchymal stem cells was transfected at ~ 70% efficiency by off the shelf cationic polymer^36^. It was, however, unknown if these modified MSC can be programmed for therapeutic functions.

The easy access to subcutaneous adipose tissue makes it an attractive alternative of bone marrow MSCs^37,38^. In the present study, we developed a process to modify AT-MSCs at high efficiency using cationic polymer in combination with this Enhancer, enabling the development of theranostic MSCs producing CDy::UPRT without the need to use virus nor the need to establish stable cell lines. Furthermore, this MSC modification process is donor and cancer agnostic, and may be useful for applications beyond the scope of this study.

## Results

### An efficient non-viral linear polyethylenimine (LPEI) based transfection method for AT-MSCs modification

**Figure 1:**
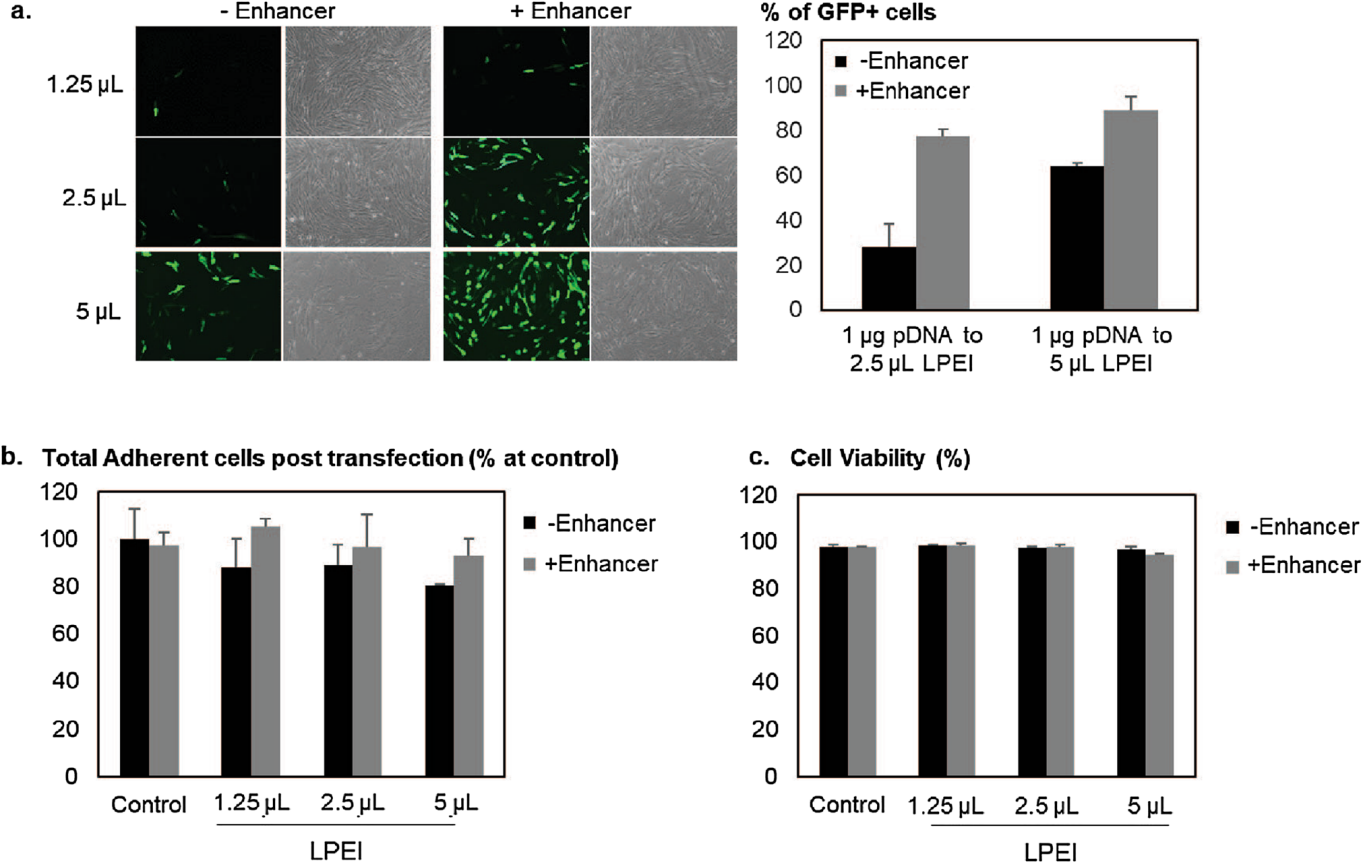
Enhancer enabled efficient LPEI based transfection in AT-MSCs. (**a**) AT-MSCs were transfected 200 ng pCMV-GFP complexed with various amount of LPEI, in the presence or absence of Enhancer. Twenty-four hours later, bright field and fluorescent images were captured. Representative images are presented. Then, cells were trypsinised, pelleted and resuspended in 1XPBS for flow cytometry analysis. Transfection efficiency was calculated as the percentage of GFP positive cells normalized to the total number of cells as quantified by FACS. Bar graph represents mean ± SD, n = 3. In the same experiment, (**b**) total number of adherent cells and (**c**) cell viability of each condition was determined with NC-3000 cell counter. Un-transfected population serves as control. Results are presented as mean ± SD, n = 3.

AT-MSCs (Age group 18–30) were transfected with a plasmid encoding GFP reporter gene in 24-well tissue culture vessels to evaluate the transfection efficiencies of LPEI and Lipofectamine 3,000 (L3K). Although, there were more cells transfected using LPEI, the number of adherent cells were less than when using L3K (Fig. S1a). While the cell viability post-transfection remained high, there was a significant reduction in adherent cell number after LPEI-mediated transfection when compare to un-transfected control. The number of adherent cells further reduced with the use of increasing amounts of plasmid DNA (pDNA) (Fig. S1b), consistent with previous observations^39–41^. Attempts to attain high adherent cell number by transfecting AT-MSCs at 200 ng of pDNA with lower amounts of polymers only resulted in significantly reduced transfection efficiency (Fig. 1).

Next, we explored the use of the Enhancer^36^ with low amount of pDNA (200 ng) and various ratios of DNA:polymer for the enhancement of transfection (Fig. 1). More than 80% of AT-MSC cells were transfected (Fig. 1a), with comparable number of adherent cells and viability to non-transfected control (Fig. 1b, c). With no apparent reduction in cell number and viability, this data suggests transfection of AT-MSCs in the presence of Enhancer does not affect cell proliferation or incur cytotoxicity. We next extended the study to include other AT-MSC isolated from another donor (Age group 31–45). Using the same protocol (at the ratio of 1 µg pDNA to 5 µL LPEI), the efficiency was as high as 80% of cells transfected with minimally 200 ng of pDNA (Fig. S2). This condition was used in further studies.

### Determination of the functionality of CDy::UPRT_AT-MSCs

**Figure 2:**
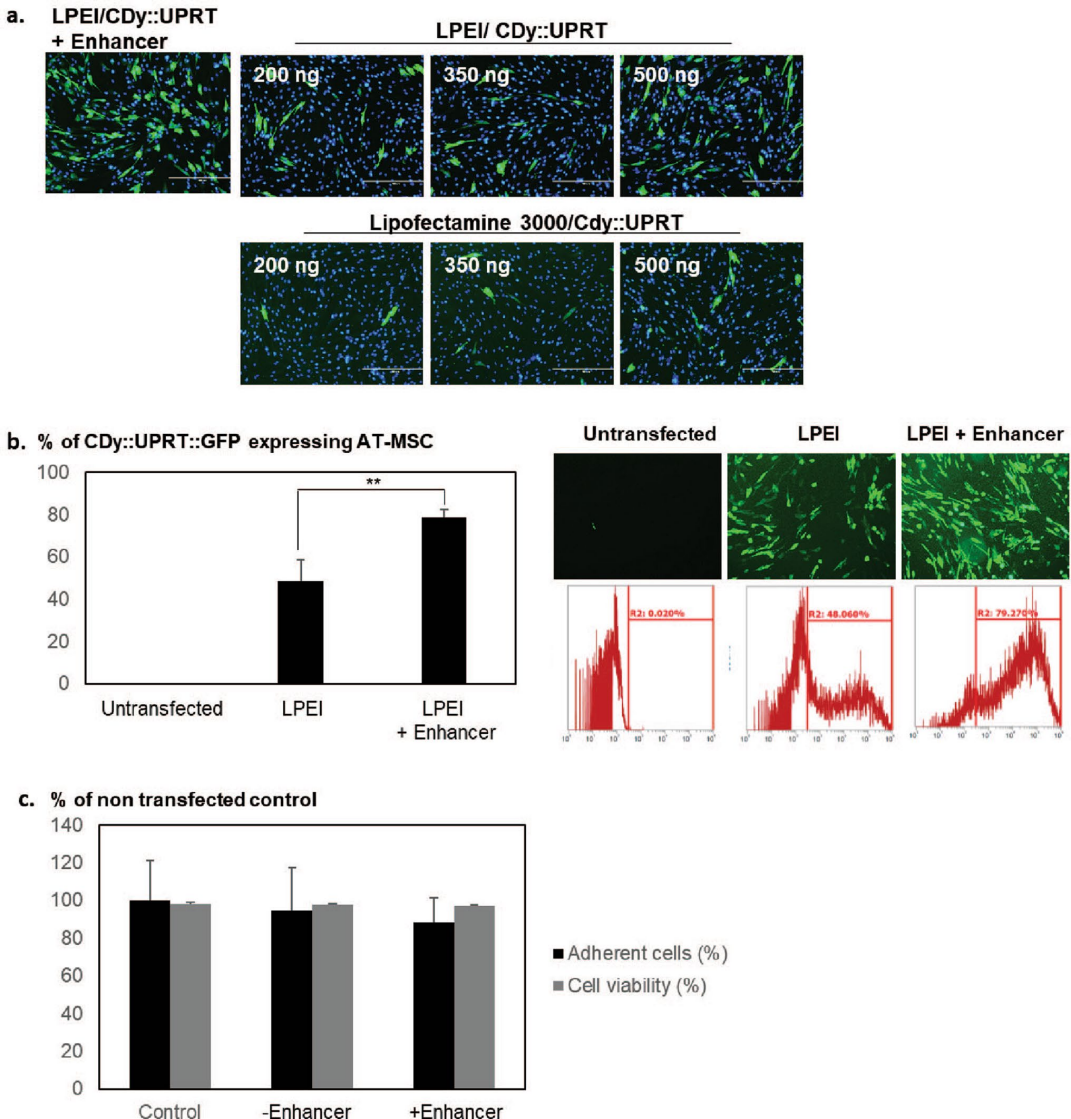
Enhancers enabled high expression of CDy::UPRT in AT-MSC. (**a**) AT-MSCs (LOT00088) cultured in 24-well vessels were transfected at various amount of CDy::UPRT expression plasmid with LPEI or Lipofectamine 3,000, using centrifugation or manufacturer’s protocol respectively. After 24 h of incubation, cells were fixed with 4% paraformaldehyde and stained for CDy (green) and nucleus (Hoechst stain, blue). Representative images are shown. Scale bar represents 400 µm. (**b**) AT-MSCs in 6 well culture vessels were transfected with 1 µg of CDy::UPRT::GFP pDNA complexed with LPEI (1 µg of DNA to 5 µL of LPEI). After centrifugation, transfection mixture was replaced with fresh media (with or without Enhancer). One day later, representative images were acquired, and cells were trypsinised for FACS analysis. Results are presented as mean ± SD (n = 4). Un-transfected AT-MSCs served as negative control for FACS analysis. Significant differences between the transfection conditions were calculated using two tailed Student’s *t*-test. **P < 0.01. (**c**) In the same experiment, total number of the cells and cell viability of each condition was determined with NC-3000 cell counter. The percentage of total adherent cells in transfected population at control (Un-transfected) was calculated. Data represented mean ± SD, n = 3.

In order to generate AT-MSCs expressing fused cytosine deaminase and uracil phosphoribosyltransferase (CDy::UPRT_AT-MSCs), cells were transfected with the CpG free expression plasmid encoding the transgene using LPEI. Based on immunocytochemistry analysis, transfection was significantly improved in the presence of Enhancer even at low amount of pDNA (200 ng). In the absence of the Enhancer, increasing pDNA amount modestly increased transfection efficiency of LPEI and Lipofectamine 3,000 (Fig. 2a), suggesting modulation of intracellular trafficking to contribute to the high transgene expression. Extending this observation, we constructed a fusion gene encoding cytosine deaminase, uracil phosphoribosyltransferase and green fluorescent protein (CDy::UPRT:GFP) for direct visualization and quantification. In the presence of Enhancer, transfection efficiency was significantly increased (~ 80%) as compared to the use of LPEI alone (~ 40%; Fig. 2b), with no significant change in viability (Fig. 2c). Notably, there was no significant difference in the anticancer efficiency of AT-MSC modified with CDy::UPRT:GFP or CDy::UPRT (Fig. S3), suggesting that addition of GFP tag did not affect CDy::UPRT function. Collectively, the results demonstrated a significant improvement in the transfection of AT-MSCs by the use of the Enhancer, which likely shares a similar mechanism in facilitating intracellular trafficking of pDNA in BM-MSC^36^.

Next, we examined the sensitivity of modified MSCs to 5FC using MTS assay. Exposure of CDy::UPRT modified AT-MSCs to 5FC reduced cell viability over time (Fig. S4a). This was to verify that the modified MSCs could express CDy::UPRT at levels sufficiently high that could induce significant cell death in the presence of prodrug. CDy::UPRT_AT-MSCs demonstrated increased sensitivity to the active cytotoxic drug 5FU (Fig. S4b). This effect was likely to be due to the activity of UPRT transgene, which catalyses the conversion of 5FU to 5-fluorouridine monophosphate^20^, consistent with previous observations^18,42^.

### Phenotypic characteristics of AT-MSC are not affected by the LPEI based transfection method

**Figure 3:**
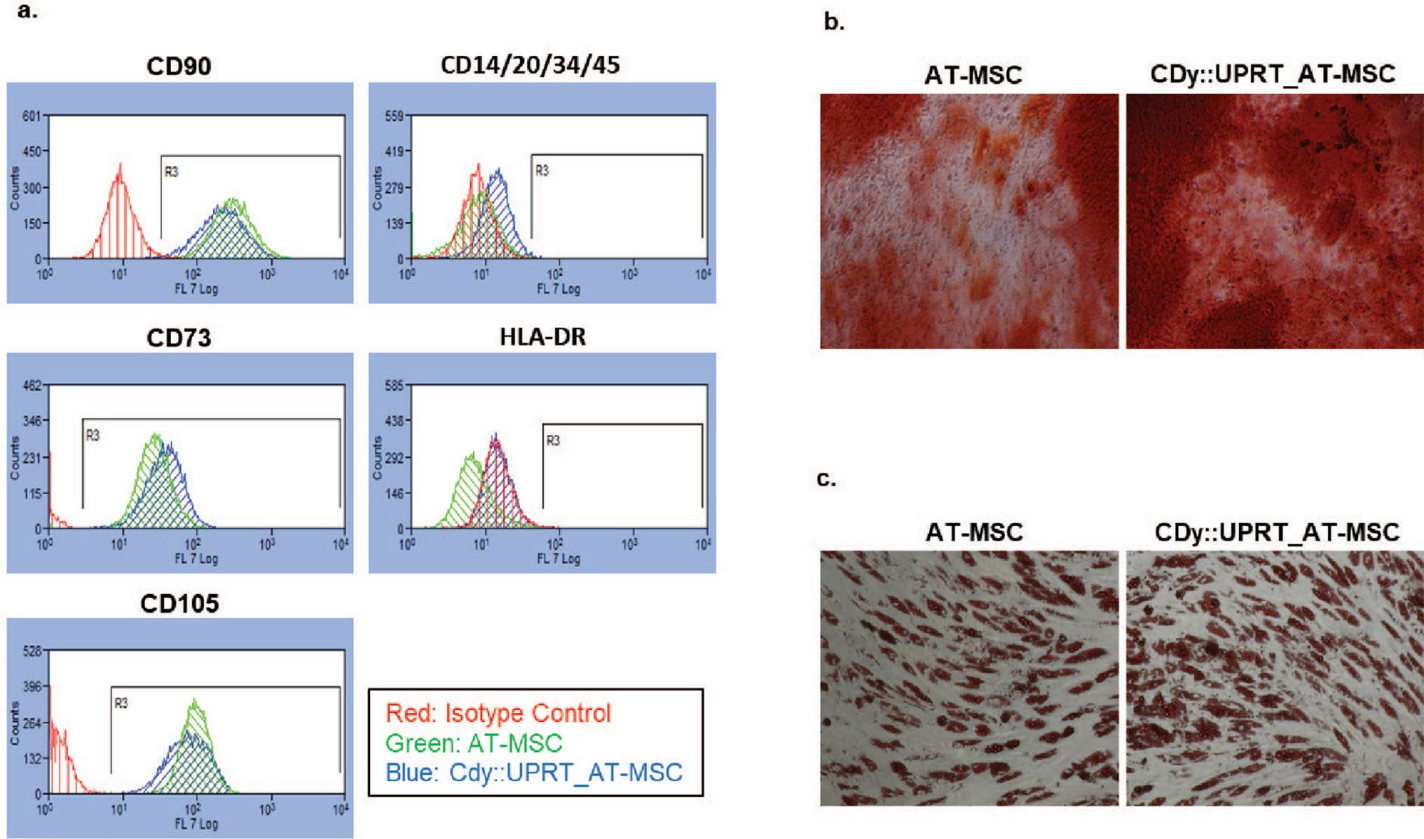
CDy::UPRT expression does not affect standard immunophenotypic profile and differentiation potential. (**a**) AT-MSCs and CDy::UPRT_AT-MSCs were labelled with fluorophore-conjugated antibodies and analysed by flow cytometry, according to the manufacturer’s instructions. Isotype antibodies serve as respective controls. Histograms demonstrated the merged profiles of isotypes (Red), un-transfected AT-MSCs (Green) and CDy::UPRT_AT-MSCs (Blue). (**b**) Un-transfected and transfected AT-MSCs were cultured in medium supplemented for osteogenic differentiation for 14 days, following manufacturer’s recommendations. At the end of incubation, cells were stained with Alizarin red S. The presence of calcium deposits stained with Alizarin red S indicates osteogenic differentiation of AT-MSCs. (**c**) Cells were cultured in medium containing components for adipogenic differentiation. Fourteen days later, cells were stained with Oil Red-O. This dye stained for oil droplets visible in the cells and was indicative of adipogenic differentiation. The images were captured at 20× magnification.

To explore the possibility that high transfection may modify the phenotype of AT-MSC, immunophenotyping of CDy::UPRT_AT-MSCs was carried out by standard FACS analysis using markers as defined by the International Society for Cellular Therapy (ISCT)^43^. The CDy::UPRT_AT-MSCs displayed identical profiles to un-transfected AT-MSCs where both cell types were found to be positive for CD90, CD73 and CD105 while negative for CD14, CD20, CD34, CD45 and HLA-DR (Fig. 3a). Expression of CDy::UPRT did not affect the differentiation capability of AT-MSCs into osteogenic (Fig. 3b) and adipogenic (Fig. 3c) lineages. Evidently, the presence of oil droplets in transfected cells indicated that the potential to differentiate into adipogenic lineage was unaffected by transfection and transgene expression (Fig. S5). In a separate study, chondrogenic differentiation was also unaffected after transfection using this method (unpublished data).

### CDy::UPRT_AT-MSCs retain tropism for cancer cell lines in vitro

**Figure 4:**
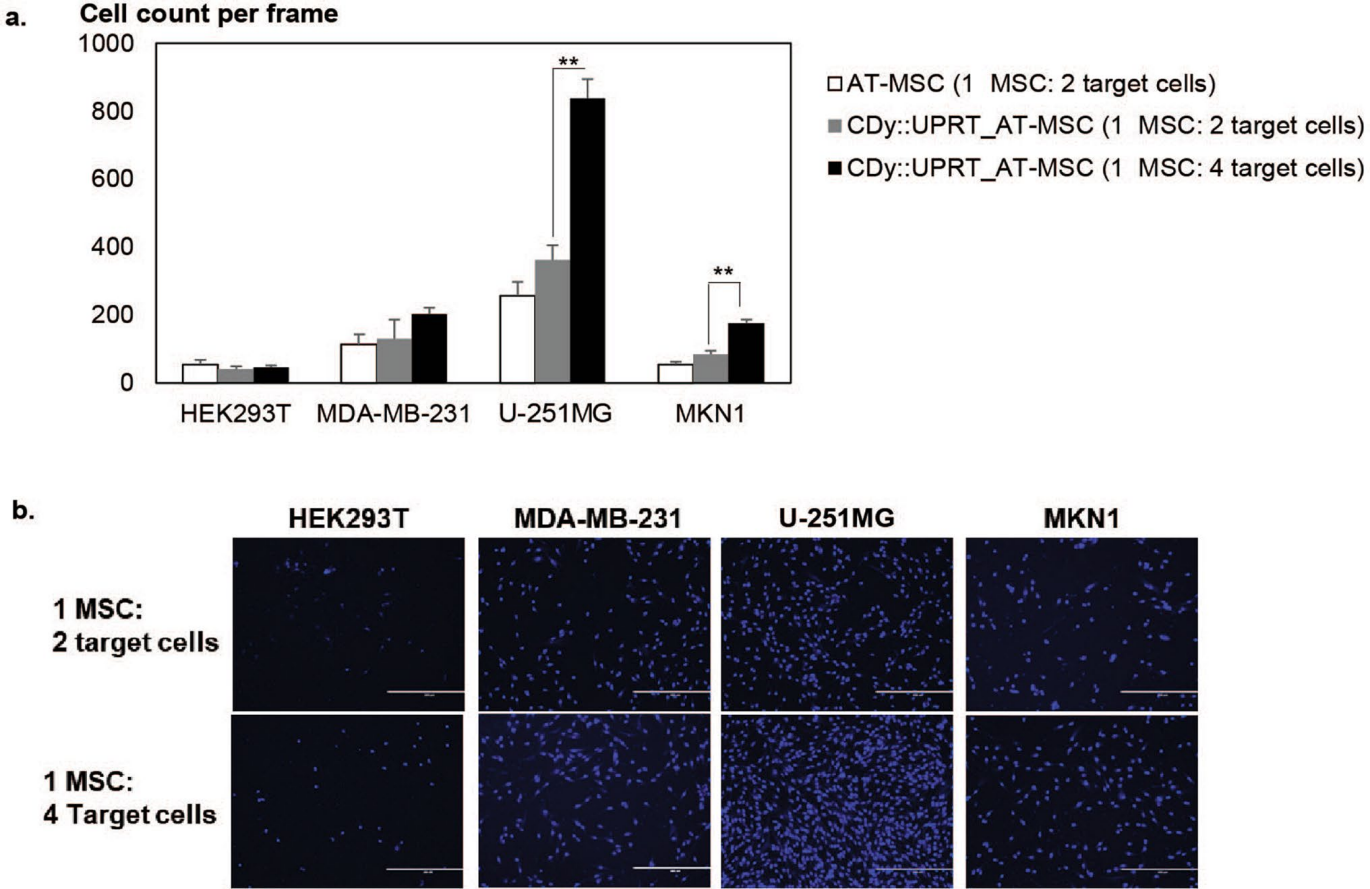
CDy::UPRT expression does not affect migration capability of AT-MSCs. (**a**) Migratory property of MSCs was evaluated using cell invasion assay. Firstly, 200,000 or 400,000 of target cells were plated in DMEM supplemented with 10% FBS. Six hours later, culture media was replaced with serum free DMEM. CDy::UPRT_AT-MSCs (transfected one day before the experiment) and un-transfected AT-MSCs were loaded onto matrigel-coated cell inserts. The inserts were transferred to the target cell cultures respectively. Twenty-four hours later, cell invasion was evaluated under microscope by taking fluorescent images of cells stained with Hoechst 33,342. Graph presents mean of migratory cells per frame (n = 3). HEK293T serves as negative control. Significant differences between the 200,000 and 400,000 target cells were calculated using two tailed Student’s *t*-test. **P < 0.01. (**b**) Representative images of migrated CDy::UPRT_AT-MSCs were shown. Scale bars, 400 µm.

The chemotactic response of AT-MSCs toward the cytokines released by cancer cells is a prerequisite for successful targeting to tumour cells^44^. Thus, it was essential to know if gene modification has altered the tropism capability of AT-MSCs to cancer cells. Here, the invasion assay was used to examine the vectorial migration of AT-MSCs through extracellular matrix in the presence of cancer cells. Invasion of AT-MSCs through extracellular matrix was significantly induced by MDA-MB-231, U-251MG and MKN1 but not HEK293T (Fig. 4a). This observation was consistent with previous reports of the chemo attraction of MSCs to cancer cells but not HEK293T^45,46^. Comparable numbers of migrated AT-MSCs and CDy::UPRT_AT-MSCs to cancer cells were observed, suggesting that the tumour homing capability was unaffected by transfection and the over-expression of CDy::UPRT. The number of CDy::UPRT_AT-MSCs that invaded through the extracellular matrix was correlated to the number of cancer cells and higher numbers of cells migrated towards U-251MG and MKN1, and lesser towards MDA-MB-231 cell lines (Fig. 4).

### CDy::UPRT_AT-MSC/5FC mediated cytotoxicity in vitro

**Figure 5:**
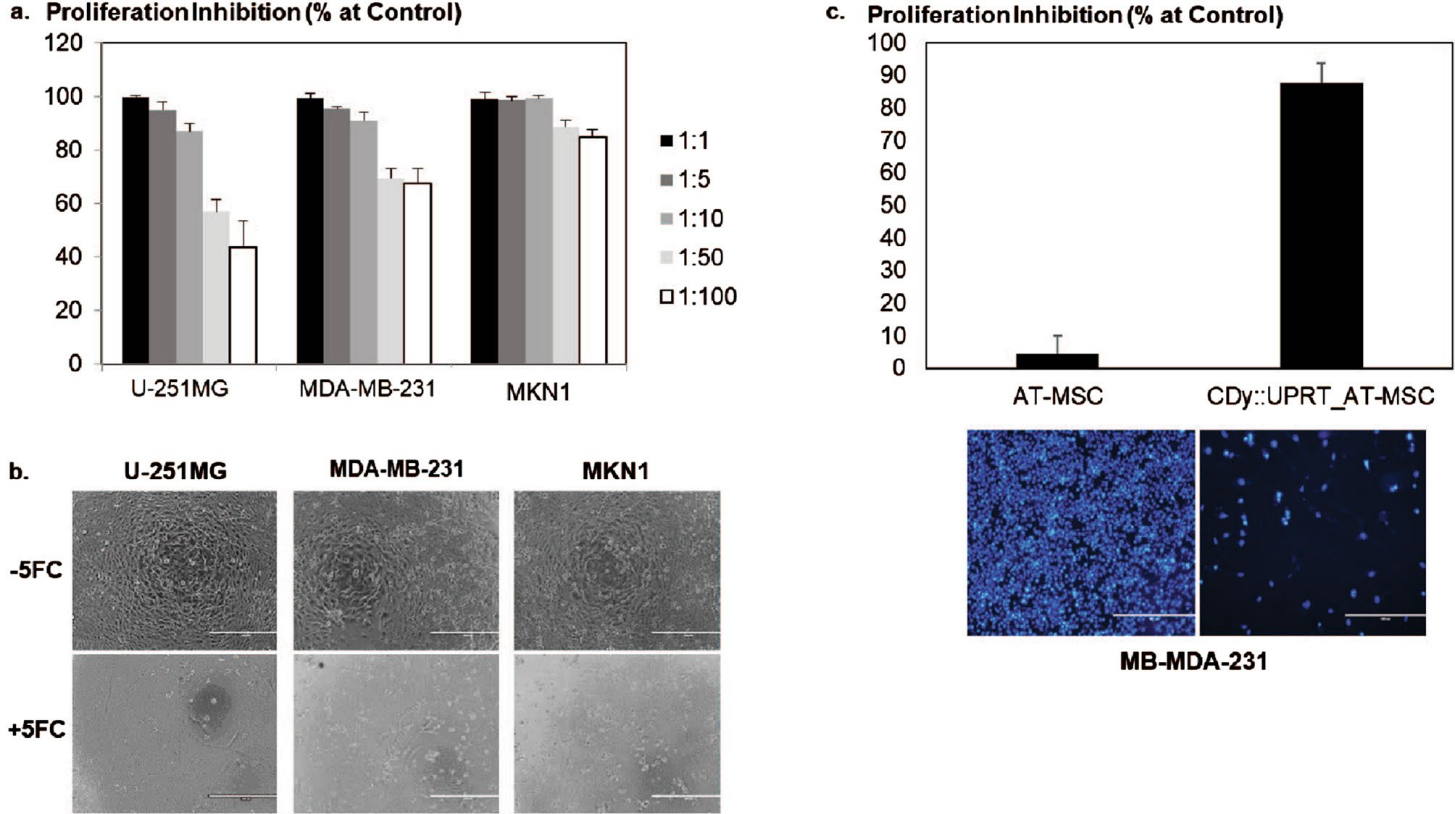
Anticancer effect mediated by CDy::UPRT_AT-MSC/5FC. (**a**) CDy::UPRT_AT-MSCs were cocultured with U-251MG, MDA-MB-231 or MKN1 in DMEM supplemented with 2% FBS, in the presence or absence of 150 µg/mL 5FC. The therapeutic cells and cancer cell lines were mixed at ratios of 1 CDy::UPRT_AT-MSC to 1, 5, 10, 50, 100 cancer cells. Five days later, proliferation inhibition in the treatment conditions was evaluated spectrophotometrically by standard MTS assay. Proliferation Inhibition was defined as 100% − (sample/control × 100%). Conditions without 5FC treatment served as controls. Data of biological quadruplicates were expressed as mean ± SD. (**b**) Bright field of the mixed cultures (1 MSC to 10 cancer cells) taken at the end of experiment. Scale bar, 400 µm. (**c**) Anticancer effect of CDy::UPRT_AT-MSCs or AT-MSCs on MDA-MB-231were evaluated by indirect coculture. Equal number of therapeutic cells and MDA-MB-231were seeded in the transwell and 24 well plates, respectively. Cells were cocultured in DMEM supplemented with 2% FBS and 5FC for 4 days. After which, transwells were removed and the remaining cells on the culture plates were stained with Hoechst 3,222. The fluorescence readout was captured with microplate reader. Proliferation Inhibition (%) was defined as 100% − (conditions with 5FC/respective conditions without 5FC × 100%). Relative fluorescence units collected from 9 areas of biological triplicate were shown as mean ± SEM. Respective images of the remaining cancer cells were shown. Scale bar represents 400 µm.

Demonstrating a cytotoxic effect of the CDy::UPRT_AT-MSCs on target cancer cells is a prerequisite for the adoption of this non-viral method of therapeutic gene modification in generating theranostic MSCs for prodrug cancer therapy. The effect of cytosine deaminase/5FC in proliferation inhibition is commonly assessed by MTS assay^47,48^. With the same approach, we first compared the anticancer effects of CDy::UPRT_AT-MSC/5FC and 5FU in glioma, breast cancer and gastric cancer cell lines (Fig. S6). At 1:1 ratio of CDy::UPRT_AT-MSC to cancer cells, the anticancer effects were comparable to the direct pharmacological effects of 5FU*.* To further examine the therapeutic potential of CDy::UPRT_AT-MSC/5FC, cells were directly co-cultured with target cancer cells at various MSC to cancer cell number ratios (Fig. 5a). Proliferation inhibition by almost 57%, 69% and 89% were observed even at co-culture ratios of 1:50 of CDy::UPRT_AT-MSC/5FC to U-251MG, MDA-MB-231, and MKN1, respectively. This ratio of mixed culture represents 2% of therapeutic cells within the cancer populations. With 10% of therapeutic cells to cancer cells, the extent of proliferation inhibition was greater than 86%. Notably, 85% proliferation inhibition was seen with only 1% of therapeutic cells in the MKN1 population. Proliferation inhibition was not observed in co-cultures without the addition of the prodrug 5FC (Fig. 5b).

Next, we explored the anticancer effects in scenarios where the therapeutic cells might not be in direct contact with the cancer cells by seeding modified MSCs in the upper chambers of transwells. Four day after exposure of MDA-MB-231 to CDy::UPRT_AT-MSC/5FC, close to 90% proliferation inhibition was observed (Fig. 5c). The anticancer efficiency of CDy::UPRT_AT-MSC/5FC in the absence of cell–cell contact was highly comparable to the direct co-culture model. Taken together, these data suggested that a potent anticancer effect can be exerted when therapeutic cells are in contact or in close proximity to the target cells. We next extended the study to compare the sensitivity of Hs738 (a non-transformed human fetal gastric/intestinal cells) and 5 gastric cancer cell lines. CDy::UPRT_AT-MSC/5FC exerted anticancer effect selectively to the gastric cancer cell lines (Fig. S7), suggesting preferential targeting of the therapeutic cells/5FC to cancerous but not non-transformed cells.

### LPEI/enhancer generates highly potent CDy::UPRT_AT-MSCs

**Figure 6:**
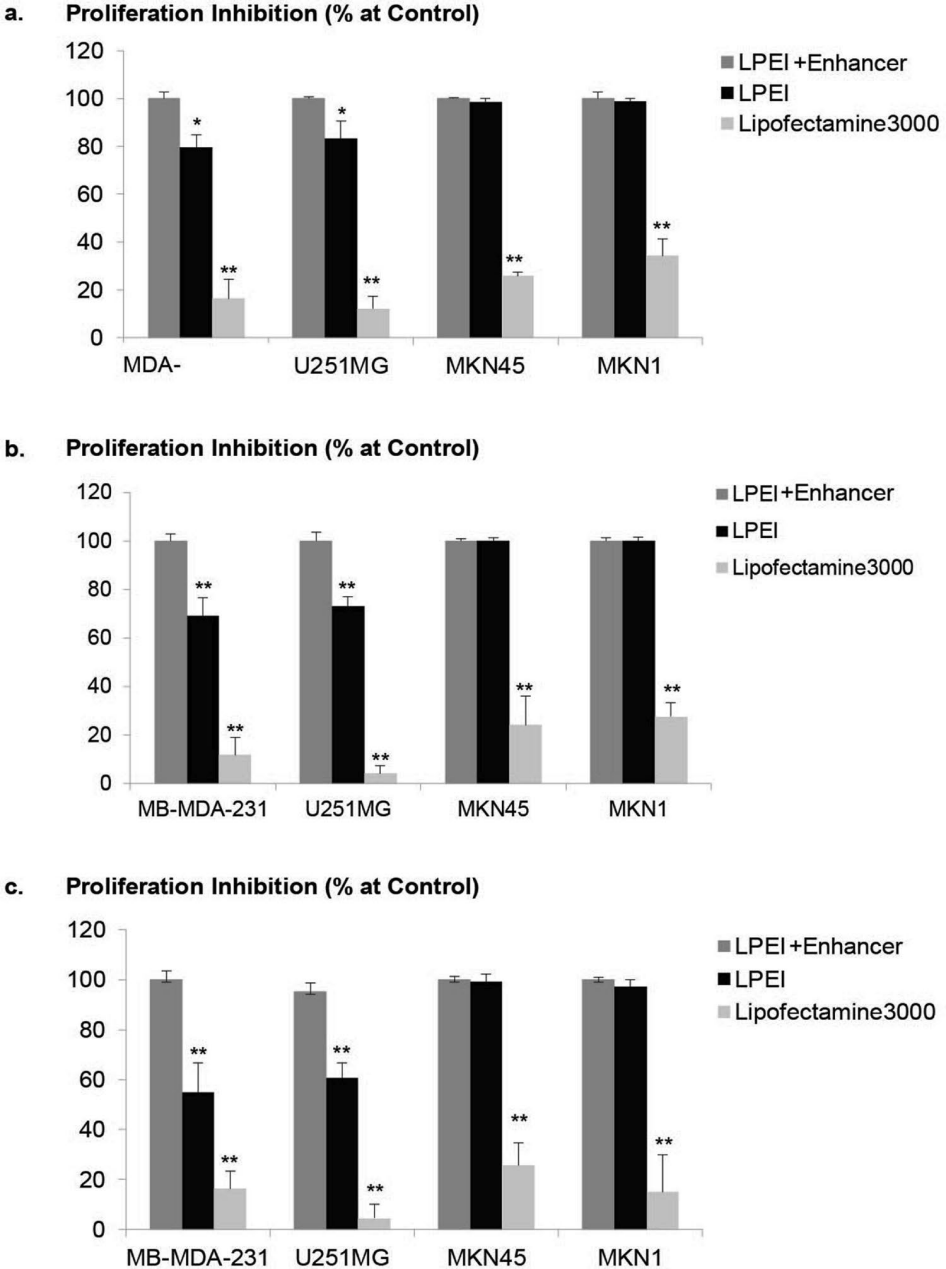
Variable anticancer effect mediated by CDy::UPRT_AT-MSC/5FC generated with different transfection methods. AT-MSCs were transfected with CDy::UPRT expression plasmid mediated by LPEI (with or without Enhancer) and Lipofectamine 3,000. One day post transfection, CDy::UPRT_AT-MSCs were cocultured with U-251MG, MDA-MB-231 or MKN1 in the presence or absence of 150 µg/mL 5FC. The therapeutic cells and cancer cell lines were mixed at ratios of 1 CDy::UPRT_AT-MSCs to 1 (**a**), 5 (**b**), 10 (**c**) cancer cells. Five days later, proliferation inhibition (%) in the treatment conditions was determined by standard MTS assay. Conditions without 5FC treatment serve as controls. Data are shown as mean ± SD (n = 4). Significant differences between conditions with LPEI + Enhancer and other methods were calculated using two tailed Student’s *t*-test. **P < 0.01.

We hypothesized that high expression of suicide gene is necessary for generating high efficacy therapeutic AT-MSCs. We compared the potencies of AT-MSCs produced by transfection with L3K and LPEI with or without the use of Enhancer (Fig. 2, Fig. S1). As expected, the anticancer efficacies of the therapeutic cells prepared with the different protocols were highly dependent on transfection efficiencies of each protocol (Fig. 6). The anticancer efficacy of CDy::UPRT_AT-MSCs generated in the presence of Enhancer significantly surpassed effects observed with cells modified with L3K. At the ratio of 1 MSC to 10 cancer cells, complete inhibition of proliferation was observed in all cancer cell lines co-cultured with CDy::UPRT_AT-MSCs generated in the presence of Enhancer. It is worthy to note that the transfection protocol using the Enhancer generated modified MSCs with similar potencies regardless of cell sources (adipocyte, bone marrow or umbilical cord derived MSCs (Fig. S8). Furthermore, we have successfully transfected MSCs with another suicide gene, Herpes Simplex Virus-1 Thymidine Kinase (HSV-TK) (Fig. S9). These data suggest the transfection workflow described herein is agnostic to MSC types and transgene.

### Prolonged expression of CDy::UPRT in AT-MSCs is possible with transient transfection

**Figure 7:**
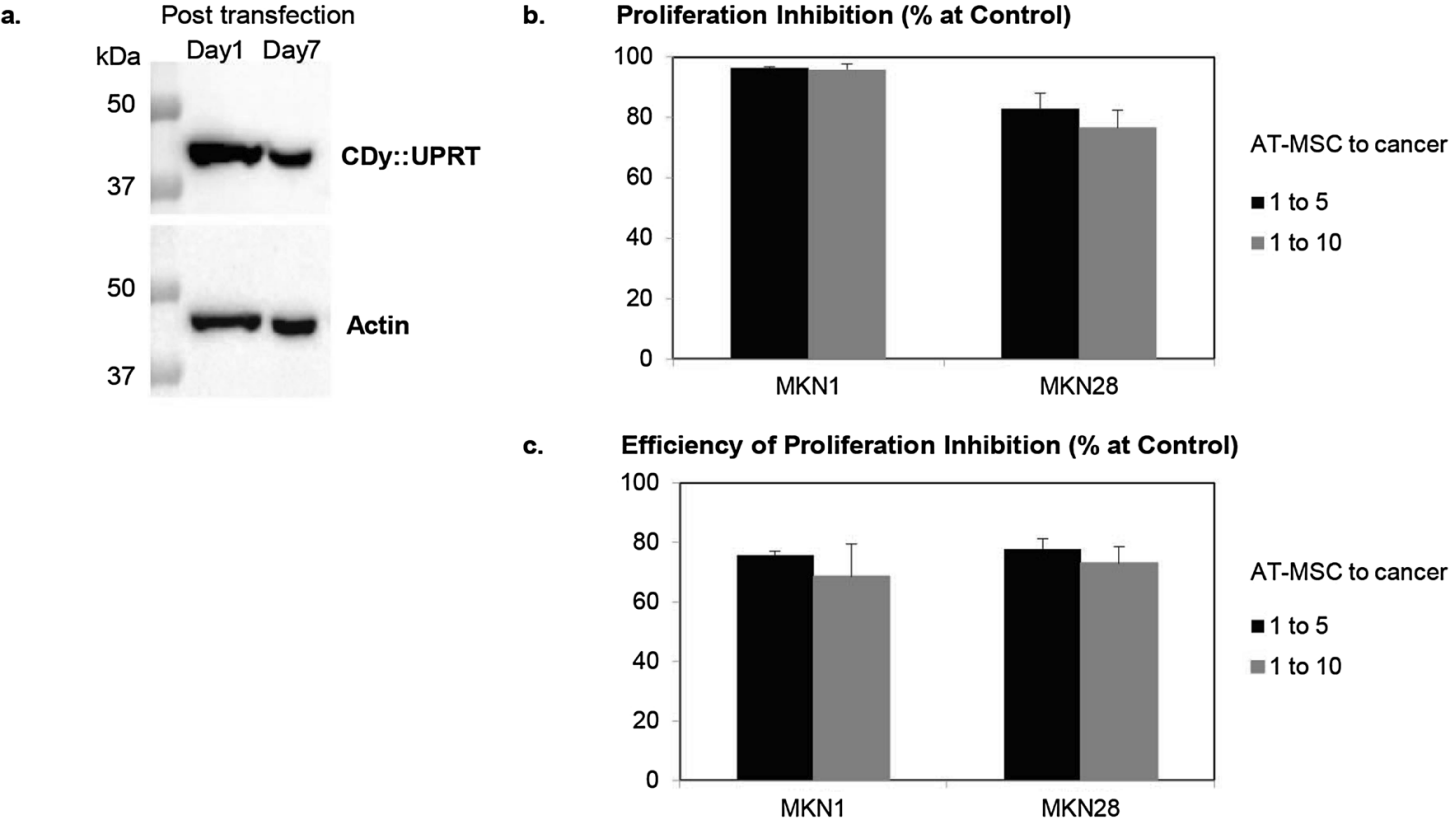
Long term expression enables sustainable anticancer efficiency of CDy::UPRT_AT-MSCs. AT-MSCs were transfected with CDy::UPRT mediated by LPEI in the presence of Enhancer. (**a**) One- or seven-day post modification, the cells were lysed for immunoblotting analysis with antibody targeting CDy. Actin was used as endogenous control for sample loading. Further information on the western blot is detailed in Fig. S12. In a parallel experiment, modified AT-MSCs were collected on day one (**b**) or seven (**c**) days post transfection. The collected cells were cocultured with MKN1 and MKN28 cell lines at the ratio of 1 MSC to 5 or 10 cancer cells, in the presence or absence of 150 µg/mL 5FC. Proliferation inhibition in the treatment conditions was evaluated spectrophotometrically by standard MTS assay after 5 days of incubation. Conditions without 5FC treatment served as controls. Proliferation Inhibition was defined as 100% − (sample/control × 100%). Graph bar represented mean (n = 4), ± SD.

To investigate the duration of expression and function of the transgene in modified AT-MSCs, the anticancer efficacy of modified MSCs collected from day 1 and day 7 post transfection were examined. Evidently, the expression of the transgene, CDy::UPRT, was significant over a period of 7 days post transfection (Fig. 7a), consistent with the observation using CDy::UPRT::GFP (Fig. S10). Comparable proliferation inhibition of cancer cells were observed with CDy::UPRT_AT-MSCs harvested on day 1 (Fig. 7b) or day 7 post transfection (Fig. 7c).

### Potential therapeutic application in targeting chemo-resistant Glioblastoma

Evidently, the cytotoxic effect of CDy::UPRT_AT-MSCs is agnostic against cancer types (Fig. 5, 6). Next, we hypothesized that this approach could potentially target chemo-resistant cancer too. A stable TMZ-resistant variant of U251-MG cell line (termed U251R) was established as described previously^49^. Proliferation inhibition of U251R in the presence of CDy::UPRT_AT-MSCs and 5FC (Fig. S11) suggested the potential use of this approach for targeting TMZ resistant Glioblastoma.

**Figure 8:**
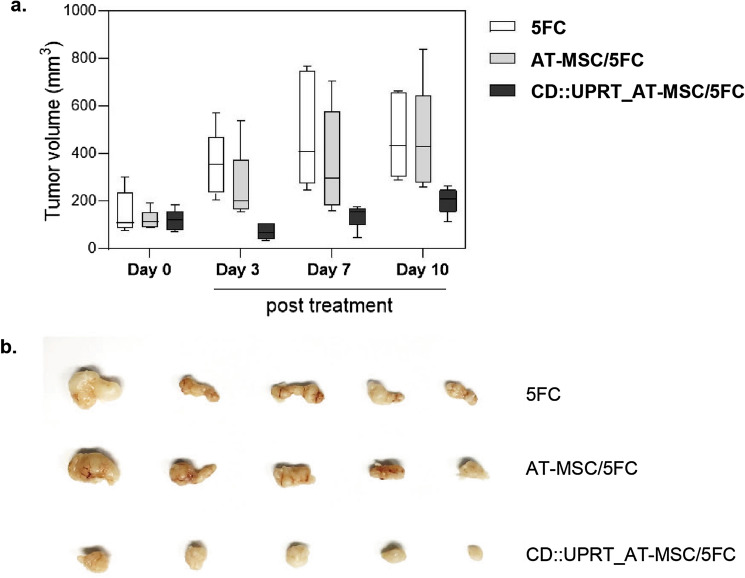
In vivo anti-tumoural effect of CDy::UPRT_AT-MSCs in the presence of 5-fluorouracil (5FU). To establish s.c tumour, 5 × 10^6^ Temozolomide resistant U-251MG cells were injected subcutaneously in dorsal flank regions. When tumour reached the target size, 1 × 10^6^ CDy::UPRT_AT-MSC or AT-MSC were injected directly to the s.c. tumour. One day later, 500 mg/kg/day of 5FC was administered daily for 4 consecutive days. The size of s.c tumour was measured with a digital calliper on day 7, 11, 15 post AT-MSC administration. Prodrug only group serves as control group. Tumour volume (mm^3^) was calculated according to the standard formula of V = (W × W × L)/2. (**a**) The box and whisker bar graph displays the distribution of tumour volume measured from n = 5 from each group. Tumour volume in treatment group (CDy::UPRT_AT-MSC/5FU) showed a statistically significant difference (P < 0.05) on day 7, 11, 15. (**b**) At the end of experiment, mice were euthanized. The tumours were extracted and fixed with 4% PFA. Image display tumours (n = 5) extracted from each group.

To provide further evidence of a therapeutic utility, CDy::UPRT::GFP_AT-MSCs were injected directly into the subcutaneous (s.c) tumour of U251R. Previous studies have shown contradictory outcomes when native MSCs were used for experimental cancer treatment^50^. To evaluate the anti-tumour effect was due to the expression of CDy::UPRT::GFP, tumour growths were monitored in 3 groups of mice. One group of animals received native MSCs and treated with 5 FC (MSC plus 5FC), another with the prodrug treatment alone (5FC), and the third group where the mice were given modified MSCs and treated with 5FC (CDy::UPRT::GFP/5FC). The tumour growth in the MSC plus 5FC group of animals did not show significant difference from the animals treated with 5FC alone. Expectedly, significant inhibition of tumour growth was observed in the CDy::UPRT::GFP/5FC group of animals (Fig. 8), where a single cycle of treatment resulted in an average of 45% reduction in tumour size (Fig. 8A). However, the tumour size in the CDy::UPRT::GFP/5FC group appeared to increase in size but yet significantly smaller than the 5FC or MSC plus 5FC groups of animals on day 7 and day 10 post treatment (Fig. 8B). This is consistent with the hypothesis that anti-tumour effect requires the MSCs to express the therapeutic suicide gene.

## Discussion

Conflicting reports have recently emerged regarding the roles of MSCs in tumour inhibition^51,52^ and growth^53–56^. These contradictions were thought to be largely due to technical differences and inherent biological heterogeneity^57^. Regardless, genetically modified MSCs is thought to offer a more suitable strategy for cancer therapy, as they are safer and more efficient than the unstable and heterogeneous naive MSCs^58,59^.

This study demonstrated the successful modification of AT-MSCs at high efficiency for the generation of theranostic AT-MSCs for prodrug cancer therapy, without the use of viruses. About half of the cell population was transfected with the commercially available polymer (PEI MAX) and the efficiency was significantly improved with low toxicity in the presence of the Enhancer (Fig. 1). This modification process did not require purification nor antibiotic selection for high expression MSCs of > 70% CD expressing cells, in line with release testing for human clinical trial^60^. Attempts to develop novel cationic polymers and lipids to modify MSCs have met with limited success due to low efficiency of transfection or high cytotoxicity^61^. Recently, a poly(β-amino-esters) (PBAE) polymer structure was reported to transfect MSCs with high efficiency and low toxicity^62^. Although the cells were well modified, the migration ability was notably affected.

For AT-MSCs to be used as targeted drug delivery vehicles for therapy,

the processes used to modify them must not change their phenotypic characteristics and behaviour, including their multipotency and their capacity for migration and invasion. No significant difference in the expression of phenotypic markers and differentiation potential of modified and native AT-MSCs was observed (Fig. 3), an essential criterion for theranostic application of the modified AT-MSCs^43^. The inherent tumour tropism is an essential feature of the homing/migration property of MSCs as a cellular vehicle for delivery of therapeutic agents^8,63^. Despite the high over expression of the transgene, the migration ability of the modified cells was comparable to the native MSCs in the presence of cancer cells in vitro (Fig. 4).

A number of GDEPT systems is being explored for cancer treatment to improve the efficacy and safety of conventional cancer chemotherapies^19^. Among the enzyme/prodrug systems tested in a recent study, the CDy::UPRT is the most effective^64^ and this has been used in stem cells based clinical trials^60,65^. In our study, CDy::UPRT modified cells inhibited the growth of MDA-MB-231, U-251MG, MKN45 and MKN1 cell lines efficiently, with as little as 10% of therapeutic AT-MSCs. Notable was that MDA-MB-231 proliferation was inhibited by ~ 90% at the ratio of 1 therapeutic MSC to 10 cancer cells (Fig. 5a). At the similar ratio, Kucerova et al. demonstrated only 40% proliferation inhibition of the same cell type when using AT-MSCs modified by retroviral transduction^66^. Yet another study reported ~ 60% reduction in cell number in the co-culture of MDA-MB-231 with virally transduced CDy::UPRT_MSCs at a ratio of 1 MSC to 4 cancer cells^67^. Furthermore, Kwon et al.^68^ and Nouri et al.^64^ using selected modified MSC, reported the inhibition of tumour growth but not regression. It is likely that the modification using the process developed herein resulted in increased payload resulting in more efficacious killing of cancer cells which is consistent with the high anticancer effects on chemoresistant glioma cells in vivo (Fig. 8). Although significantly less than the control groups (5FC alone or native MSC with 5FC), the apparent increase in tumour size over a period of time with a single treatment may be further improved with multiple treatment cycles and this is the subject of an ongoing investigation.

MSCs mediated CD/5FC treatment has been suggested as a strategy to overcome the systemic toxicity of 5FU^14,68^. In the in vivo study, we did not observe significant change in the weight of subjects or other direct side-effects (data not shown), consistent with other studies^14,68^. Because of the alleviation of systemic toxicity, repeated injection of CD-MSC is possible to enhance the antitumour activity. Additionally, it is noteworthy that the therapeutic MSCs itself is sensitive to the CDy::UPRT/5FC system (Fig. S4), thus limiting the survival of the therapeutic cells; fulfilling a crucial requirement of a ‘hit and run’ strategy, leaving no trace of the cellular vehicle^8^.

Depending on the route of administration and location of the tumour, it is anticipated that 1 to 4 days are required for MSCs to biodistribute to residual tumour and home to distant foci of tumour^69^. In the recent clinical trial on advanced gastrointestinal cancer, patients were given three treatment cycles with modified MSCs followed by the prodrug administered 48–72 h later^65^. In parallel, 4 days after the administration of modified neural stem cells, prodrug (5FC) was administered and the modified cells were functional during the entire 7-day course of 5FC^60^. Hence, it is conceivable that the transiently transfected AT-MSCs with prolonged expression of CDy::UPRT (Fig. 7) should be effective over the duration of a treatment regime.

In order to reduce toxicity due to prolonged exposure to the polyplex, a low speed spinning step was used^36,70^, which may limit the production of theranostic AT-MSC in large scale. Process design and optimization is underway for scalability in production by precluding the low speed centrifugation step.

In conclusion, we herein described an in vitro, non-viral process for engineering theranostic AT-MSCs for GDEPT with high efficiency and high cell viability using an off-the shelf cationic polymer. We showed that, despite the high over expression of the transgene, the phenotypic characteristics and migration ability of the modified cells were comparable to the native MSCs. These cells were highly efficient in inhibiting proliferation of cancer cells in vitro. Hence, this process to modify AT-MSCs constitutes an effective and safe alternative to viral transduction for stem cell-based cancer therapy and may be useful for applications beyond the scope of this study.

## Methods and materials

### Cell culture

Human adipose tissue derived mesenchymal stem cells (AT-MSCs, RoosterBio) was isolated from female donor (LOT00088, age group 18–30). The procurement of human AT-MSCs was carried out in accordance with relevant guidelines and regulations. The sample is de-identified and commercially available. AT-MSC was maintained in the hMSC High Performance Basal Media (Roosterbio). Breast cancer cell line MDA-MB-231 (HTB-26, ATCC), and primary human dermal fibroblast (ATCC, PCS-201-012), were cultured and maintained according to manufacturer's instruction. Glioma cell line U-251MG was kindly provided by Paula Lam (Duke NUS Medical School). U-251MG cell line was cultured in DMEM (Dulbecco Modified Eagle Medium) supplemented with 10% Fetal Bovine Serum (FBS, Biowest). Gastric cancer cell line MKN1 and MKN28 were kindly provided by Dr. Yong Wei Peng (National University Cancer Institute, Singapore). The gastric cancer cell lines were cultured in RPMI (Roswell Park Memorial Institute medium, Thermo Scientific), supplemented with 10% FBS. Cells were kept at 37 °C in humidified atmosphere and 5% CO2.

### Construction of CpG free expression plasmid containing CDY::UPRT

Plasmid DNA (pDNA) expressing fused cytosine deaminase and uracil phosphoribosyltransferase (4265 bp pSELECT-zeo-FcyFur) was purchased from InvivoGen. Construction of CpG free expression plasmid of CDY::UPRT was performed by cross-lapping in vitro assembly (CLIVA) cloning techniques as described by Zou and colleagues^71^. Briefly, Lucia in the plasmid pCpGfree-Lucia (InvivoGen) was replaced with CDy::UPRT using pSELECT-zeo-FcyFur as the template in polymerase chain reaction (PCR). All pDNA were propagated in Escherichia coli GT115 strain (InvivoGen) under the selection of Zeocin. The plasmids were purified with E.Z.N.A. endo-free plasmid maxi kit according to manufacturer’s instruction (Omega Bio-tek).

### Transfection procedure

Transgene of interest was introduced into AT-MSCs at passage 3–6. For each well (6-well plate format), 1 mg/mL of LPEI (PEI MAX, Polyscience) was added to pDNA in serum free DMEM at different ratio of pDNA and LPEI. The mixture, at a total volume of 100 µl, was incubated at room temperature for 15 min. The pDNA:LPEI ratio was calculated according to the amount of 1 µg pDNA: volume of 1 mg/mL of LPEI. LPEI/pDNA complex was then added to serum free DMEM medium (1:20) to prepare for the transfection mixture. The culture media was removed and replaced with the transfection mixture, followed by mild centrifugation at 200 g for 5 min. After centrifugation, the transfection mixture was removed and replaced with complete media, with or without supplementation of transfection Enhancer. The final concentrations of the Enhancer were 10 µg/mL of DOPE/CHEMS (9:2 molar ratio) (Polar Avanti Lipid) and 1.25 µM Vorinostat (SAHA, Bio Vision). Cells were incubated for 24 h before analysis.

### Expression analysis

Flow cytometry, western blot and immunocytochemistry were performed as previously described^36^. Briefly,

#### Flow cytometry

For flow cytometry analysis (FACS), the cells were trypsinised, centrifuged and re-suspended in PBS. Cell clumps were removed by filtering through 40 mm mesh. Percentage of fluorescence positive cells was quantified by Attune NxT Flow Cytometer system (ThermoFisher Scientific) and the raw data was analysed using Invitrogen Attune NxT software (ThermoFisher Scientific). At least 10,000 cells were analysed per sample.

#### Imaging

Cell images were taken with EVOS FL Cell Imaging System (ThermoFisher Scientific) equipped with three fluorescent light cubes for viewing of DAPI (Ex357/Em447), GFP (Ex470/Em510) fluorescence.

#### Western blot

Cells were harvested and lysed with lysis buffer at various duration post transfection. Cell lysis buffer consisted of 150 mM sodium chloride, 1.0% NP-40, 50 mM Tris pH 8.0) supplemented with protease inhibitor cocktail (Roche). The whole cell lysate (20 µg) were resolved on 10% polyacrylamide sodium dodecyl sulphate gels and analysed by immunoblotting technique with sheep anti-CDy (PA185365, ThermoFisher Scientific) and monoclonal anti-β-Actin (A2228, Sigma-aldrich), respectively.

#### Immunocytochemistry

Un-transfected and CDy::UPRT producing AT-MSC (1 day post transfection) were fixed with 4% formaldehyde in 1xPBS for 20 min at room temperature. Subsequently, cells were permeabilized in 0.5% Triton-X100 in 1xPBST. The samples were treated with 5% BSA in 1xPBST for 30 min at 37 °C. The cells were then incubated with primary antibodies against CDy in 0.1% TritonX-100/1% BSA/1xPBST at 37 °C for 2 h and washed three times in 1xPBST. Then, the samples were incubated with Alexa Fluor 488 donkey anti-sheep fluorescent secondary antibody (A11015, ThermoFisher Scientific) diluted 1:200 in 0.1% Triton X-100/1% BSA/1xPBST for 2 h at 37 °C. The cells were washed three times in 1xPBST. Image acquisition was performed using the EVOS FL Cell Imaging System. All images were taken with identical optical settings.

### Characterization and differentiation potential of CDy::UPRT producing AT-MSCs

To examine the phenotype of CDy::UPRT producing AT-MSCs, cells were labelled with MSC Phenotyping Kit consisting of antibodies CD73, CD90, CD105, CD14, CD20, CD34, CD45, and HLA-DR (Miltenyi Biotech) according to manufacturer’s instructions. After which, expression of the markers was analysed with FACS. High quality MSC population consist of > 95% CD90, CD105, and CD73 positive cells. The population expressing CD14, CD20, CD34, CD45, and HLA-DR should be < 1%^43^. The multipotency of AT-MSCs was confirmed by its differentiation capacity into osteogenic and adipogenic lineage^43^. Differentiation of AT-MSCs was induced with StemPro Osteogenesis Differentiation Kit and StemPro adipogenesis Differentiation Kit (ThermoFisher Scientific). Un-transfected AT-MSCs were used as control. The phenotype and differentiation potential of CDy::UPRT producing AT-MSCs should be similar to the un-transfected AT-MSC.

### In vitro drug susceptibility

Quadruplicates of AT-MSC, MKN1, MKN45, MDA-MB-231 (10,000 cells per well) and U-251MG (5,000 cells per well) for each treatment were plated into 96-well plates. Twenty-four hours later, culture medium was replaced for medium containing various concentration of 5-Fluorocytosine (5FC, InvivoGen) or 5-Fluorouracil (5FU, InvivoGen). One to five days later, plates were subjected to the CellTiter 96 Aqueous One Solution Cell Proliferation Assay (Promega). The colorimetric read out was measured spectrophotometrically at 490 nm. Results were expressed as the percentage of cell viability, in relative to cells in condition without 5FC or 5FU (set to 100%).

### Anticancer efficacy of CDy::UPRT producing AT-MSCs in vitro

#### Direct co-culture

Quadruplicates of gastric cancer cell lines and breast cancer cell line (5,000 cells) and U-251MG (2,000 cells) were plated in 96-well plates. Five hours later, various numbers of either un-transfected or CDy::UPRT-producing AT-MSCs at the ratios of 1 AT-MSC to 1, 5, 10, 50 and 100 cancer cells were added to the cancer cells. One day later, the culture media was replaced with DMEM supplemented with 2% FBS, with or without 5FC (0–150 µg/mL). Five days later, cell viability was measured by proliferation assay. Conditions without 5FC was set to 100%.

#### Indirect coculture

MDA-MB-231cells were plated on 24-well plate (5 × 10^4^ cells per well). AT-MSCs or CDy::UPRT_AT-MSCs (5 × 10^4^ cells per well) were plated on transwell (Corning, C05/3,422). After 6 h of cultivation, inserts with therapeutic cells were transferred into the wells with MDA-MB-231cell line, with or without 5FC. Cytotoxic effect was evaluated after 4 days of incubation. Transwells were removed and culture media was replaced with 1XPBS containing 1 µg/mL of Hoechst 3,222. Stained cells were analysed using Synergy H1 microplate reader at excitation and emission wavelength of 358 nm and 461 nm, respectively. With gain setting at 80, Relative Fluorescence Unit (RFU) at 9 areas of the cell culture were recorded. Proliferation inhibition after treatment will be calculated relative to the control (coculture of untransfected AT-MSC and MDA-MB-231 cells).

### Anticancer efficacy of CDy::UPRT producing AT-MSCs in vivo

Five to six-week old female nude mice were purchased from InVivos and used for the in vivo studies under National University of Singapore Institutional Animal Care and Use Committee (IACUC) approved protocol (R18-1,383). All methods were carried out in accordance with relevant guidelines and regulations. Mice were anesthetized by isoflurane inhalation and 5 × 10^6^ Temozolomide resistant U-251MG cells suspended in 100 μL DMEM (50% Matrigel) were injected s.c. in dorsal flank regions. The growth of tumour was monitored by digital caliper. When tumours measured an average volume of 80–200 mm^3^, treatment was started. All mice were randomly distributed into 3 groups each containing 5 mice. Prodrug control group received daily injections of prodrug. Cell control group received intratumoural injection of 1 × 10^6^ MSCs plus daily injections of prodrug. Treatment group received intratumoural injection of 1 × 10^6^ CDy::UPRT_AT-MSCs plus daily injections of prodrug. Modified or non-modified MSC were administrated intratumourally on day 0 (single dose). One day later, mice received i.p. administration of 500 mg/kg of 5FC for 4 consecutive days. Before cell injection (Day 0) and Day 7, 11 and 15 after MSC administration, tumour sizes and body weights were measured.

### Cell invasion assay

The tumour tropism of AT-MSCs was determined using BD Biocoat matrigel invasion chambers (BD Biosciences). Cancer cell lines or HEK293T cells were loaded in the lower well of the 24-well plates. Twenty four hours later, un-transfected and CDy::UPRT-producing AT-MSCs in serum-free DMEM were added onto the invasion chambers. Lower wells were washed with 1XPBS, filled with serum free DMEM, for the invasion assay. After 24 h incubation, non-invading cells and matrigel were removed from the top chamber of the insert. Invaded cells were stained with Hoechst 33,342 (ThermoFisher Scientific) and photographed through the imaging system. Number of cells in 3 frames were counted.

### Statistical analysis

Where Student's *t*-test, was used, an unpaired two-tailed test was used, with the assumption that changes in the readout are normally distributed.

## Supplementary information

Supplementary information.

## Abbreviations

MSC: Mesenchymal stem cell
PEI: Polyethylenimine
HDAC6i: Histone deacetylase 6 inhibitor
CDY::UPRT: Fused yeast cytosine deaminase::uracil phosphoribosyltransferase
CD: Cytosine deaminase
GDEPT: Gene-directed enzyme prodrug therapy
UPRT: Uracil phosphoribosyl-transferase
FUMP: 5-Fluorouridine monophosphate
5FC: 5-Fluorocytosine
5FU: 5-Fluorouracil
DPD: Dihydropyrimidine dehydrogenase
OPRT: Orotate phosphoribosyltransferase
GFP: Green fluorescence protein
AT-MSC: Human adipose tissue derived MSC
DMEM: Dulbecco Modified Eagle Medium
FBS: Fetal Bovine Serum
pDNA: Plasmid DNA
MOI: Multiplicity of infection
HSV-TK: Herpes Simplex Virus-1 Thymidine Kinase
S.c: Subcutaneous
i.p: Intraperitoneal
