# Supplementary material for: A highly efficient non-viral process for programming mesenchymal stem cells for gene directed enzyme prodrug cancer therapy
Source: Sci Rep. 2020 Aug 31;10:14257. doi: 10.1038/s41598-020-71224-2 (PMC7458920; doi:10.1038/s41598-020-71224-2)
Supplement: Supplementary file 1 — Supplementary information. [file 41598_2020_71224_MOESM1_ESM.pdf]

A highly efficient non-viral process for programming Mesenchymal Stem Cells for Gene Directed Enzyme Prodrug Cancer Therapy

Yoon Khei HO[1]*, Jun Yung WOO[1], Geraldine Xue En TU[1], Lih-Wen DENG[1], Heng-Phon TOO[1]*

[1]Department of Biochemistry, National University of Singapore, 119260, Singapore.

*To whom correspondence should be addressed. Tel: +65 65163687; Fax: +65 64789561; Email: bchhyk@nus.edu.sg (Y.K. Ho), Email: bchtoohp@nus.edu.sg (H.P. Too)

Supplementary Figures

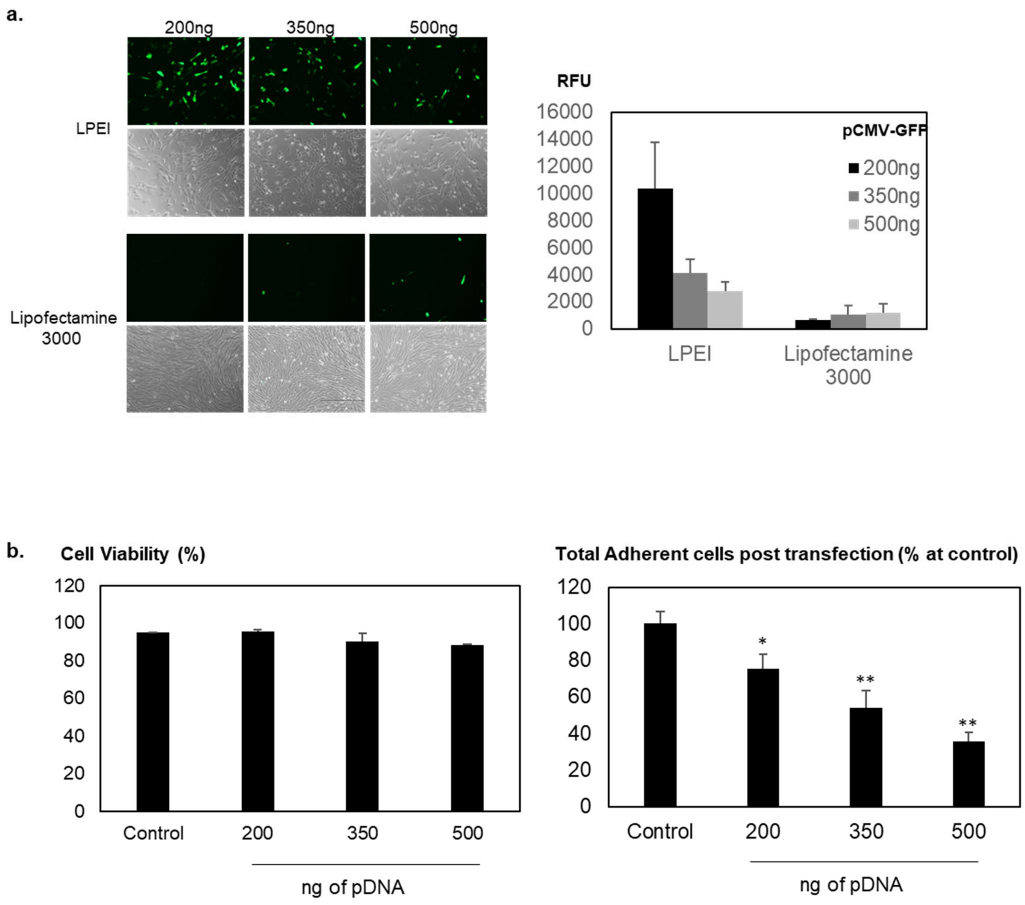

Figure S1. Reduction in cell number with increasing DNA amount (a) LPEI/ pCMV-GFP polyplex or Lipofectamine 3000/pCMV-GFP lipoplex were prepared at various amount of pDNA. AT-MSCs were transfected by LPEI (1 µg pDNA to 10 µL LPEI) or Lipofectamine 3000 following centrifugation protocol or manufacturer's instruction respectively. Relative fluorescence unit (RFU) of the GFP expression was measured spectrophotometrically (Ex475/Em509) at nine areas of each biological replicates (n=3). Graph represents mean of RFU $\pm$ SEM. **(b)** After LPEI_transfection, adherent cells were trypsinised and stained with Propidium Iodide (PI) and Hoechst 33342 (H33342). The cell viability and total adherent cells were determined with NC-3000 cell counter, according the manufacturer's protocol. Un-transfected population serves as control. Cell viability (%) represents percentage of PI negative cells. Percentage of total adherent cells were calculated in relative to control, which was set at 100 %. Data are expressed as mean $\pm$ SD of experiment performed in biological triplicate. Significant differences between control and transfected samples were calculated using the two tailed student's t-test. **, $p < 0.05$.

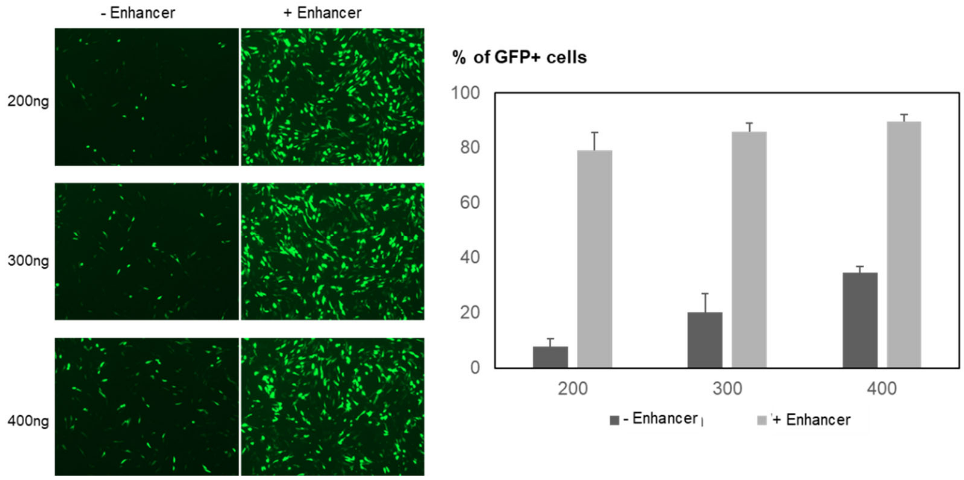

Figure S2. High transfection efficiency in AT-MSC isolated from different donor. AT-MSC was isolated from female donor, age group 31-45 (LOT00061, Roosterbio). LPEI/pCMV-GFP polyplexes were prepared at various amount of pDNA at the ratio of 1μg pDNA to 5 μL LPEI. Representative images were acquired 24 h later. Then, cells were trypsinised, pelleted and resuspended in 1XPBS for flow cytometry analysis. Transfection efficiency was calculated as the percentage of GFP positive cells normalized to the total number of cells as quantified by FACS. Bar graph represents mean ± SD, n = 3.

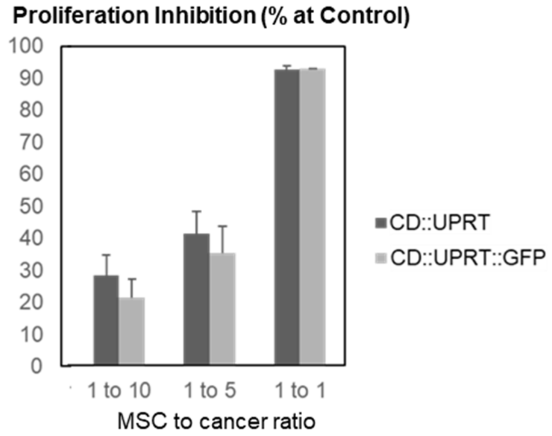

Figure S3. Comparable anticancer efficiency of MSC modified with CDy::UPRT and CDy::UPRT::GFP. MSC (200,000 cells) were transfected with 1 μg of CDy::UPRT or CDy::UPRT::GFP, in the presence of Enhancer. One-day post transfection, U-251MG cells were co-cultured with CDy::UPRT_MSC at a ratio of 1:1, 5, or 10 (MSC:cancer cells) in DMEM supplemented with 2 % FBS, with or without 100 μg/mL 5FC. Five days later, proliferation inhibition in the treatment conditions was evaluated spectrophotometrically by standard MTS assay. Conditions without 5FC treatment serve as control, which was set as 0 %. Proliferation inhibition (%) was calculated in relative to control. Data collected from quadruplicates are expressed as mean ± SD.

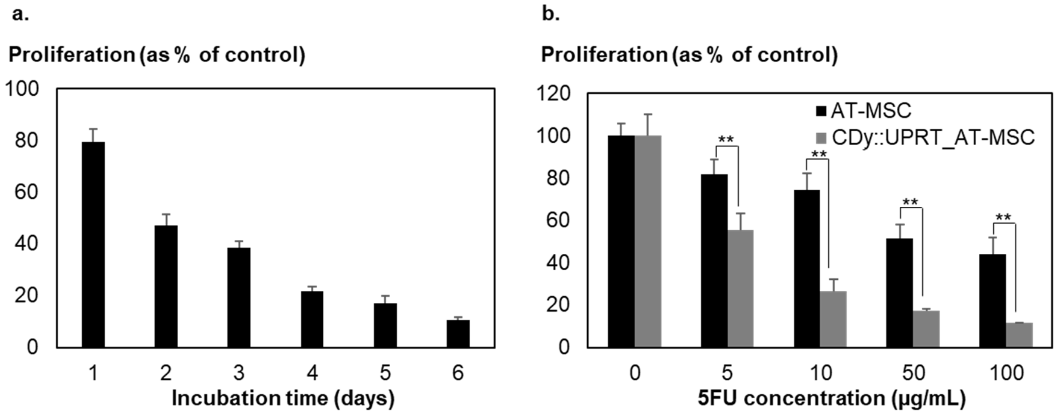

Figure S4. CDy::UPRT expression renders AT-MSCs sensitive to 5FC and 5FU. (a) CDy::UPRT_AT-MSCs were treated with 150 µg/mL of 5FC for the indicated durations. The cell viability at each time point was measured by standard MTS assay. At various time points, conditions without 5FC treatment was set at 100 %. **(b)** Sensitivity to 5FU was compared between un-transfected and modified AT-MSCs after 5 day of culture in the presence of 5-100 µg/mL 5FU. MTS assay were used to determine cell viability post treatment. Conditions without 5FU treatment was taken as 100 %. Data are presented as mean ± SD (n = 4). Significant differences in proliferation between AT-MSCs and CDy::UPRT_AT-MSCs were calculated using the two tailed student's t-test. **, $p < 0.005$.

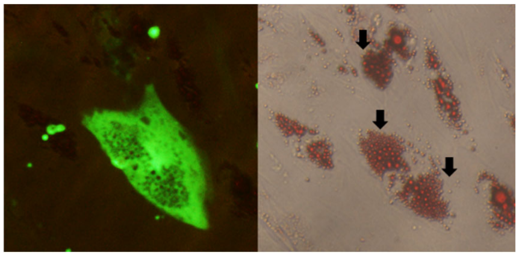

Figure S5. Adipogenic differentiation of CDy::UPRT::GFP expressing AT-MSC. AT-MSCs were transfected with CDy::UPRT::GFP in the presence of Enhancer. One-day post transfection, the media was replaced with adipogenic differentiation media. Fourteen days later, cells were stained with Oil Red-O. Modified AT-MSCs as indicated with GFP expression display visible oil droplets, suggesting multipotency of AT-MSC remain unchanged post transfection.

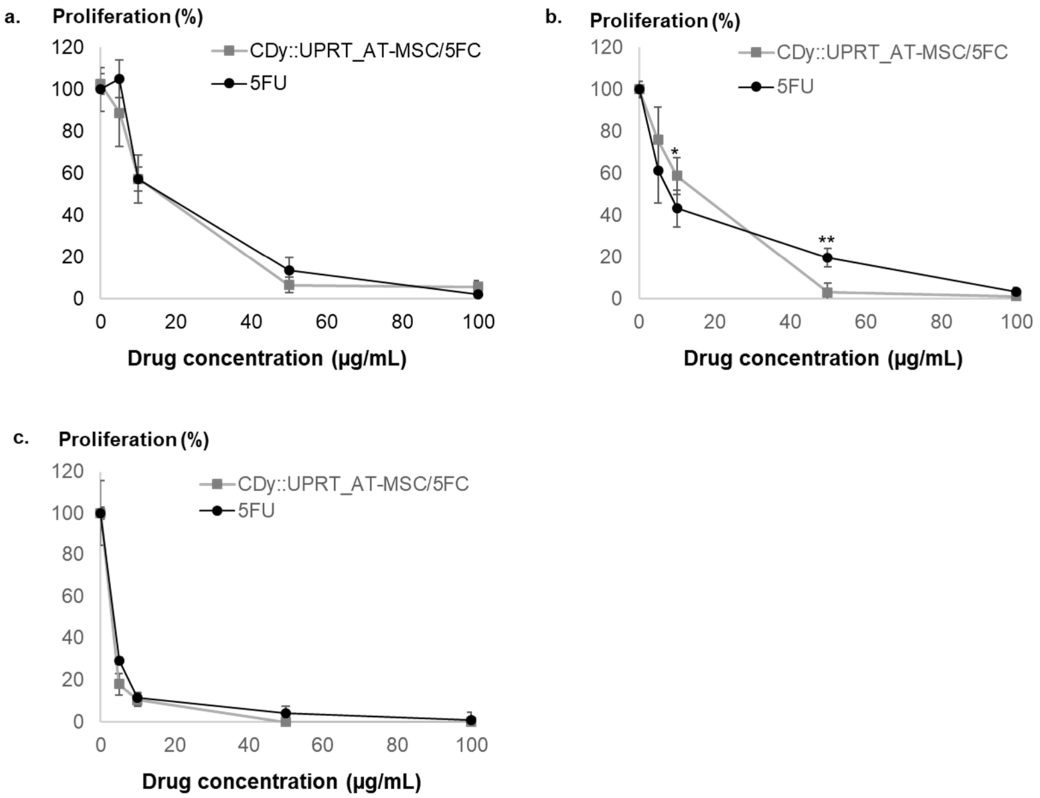

Figure S6. Comparable anticancer efficiency of CDy::UPRT_AT-MSC/5FC and 5FU. The anticancer effect was evaluated in **(a)** U-251MG, **(b)** MDA-MB-231 and **(c)** MKN1. The anticancer effect of CDy::UPRT_AT-MSCs in combination with 5FC was analysed by coculture of equal number of CDy::UPRT_AT-MSCs and cancer cell lines (2,000 U251-MG, 5,000 MDA-MB-231 and 5,000 MKN1). One day later, the culture media was replaced with DMEM supplemented with 2 % FBS and 5FC (5, 10, 50, 100 μg/mL). On the other hand, 4,000 U-251MG, 10,000 MDA-MB-231 and MKN1 were seeded 24 h before 5FU treatment. The cell lines were treated by 5, 10, 50, 100μg/mL of 5FU in DMEM supplemented with 2 % FBS. After 5 days of incubation, the cell proliferation was evaluated qualitatively by standard MTS assay. Conditions without treatment of 5FC and 5FU serve as negative controls that were set as 100 %. Graph represents mean ± SD, n = 4.

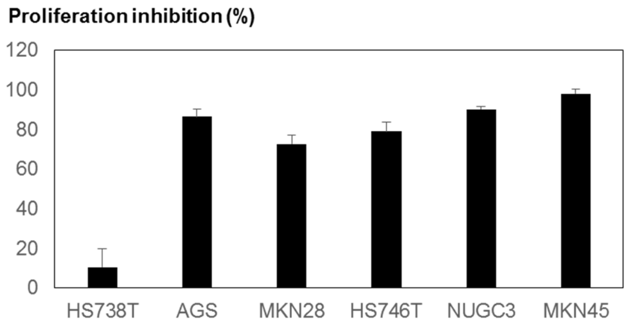

Figure S7. Selective proliferation inhibition of CDy::UPRT_AT-MSC/5FC on cancer cell lines. CDy::UPRT_AT-MSCs were cocultured with HS738T (ATCC, CRL-7869), AGS, MKN28, HS746T, NUGC3 and MKN45 (provided by Dr. Yong Wai Peng). The mixed cultures were incubated DMEM supplemented with 2 % FBS, in the presence or absence of 150 μg/mL 5FC. The therapeutic cells and cancer cell lines were mixed at ratios of 1 CDy::UPRT_AT-MSC to 10 cancer cells. Five days later, proliferation inhibition in the treated conditions was evaluated spectrophotometrically by standard MTS assay. Conditions without 5FC treatment serve as controls. Graph bar represents mean ± SD, n=4.

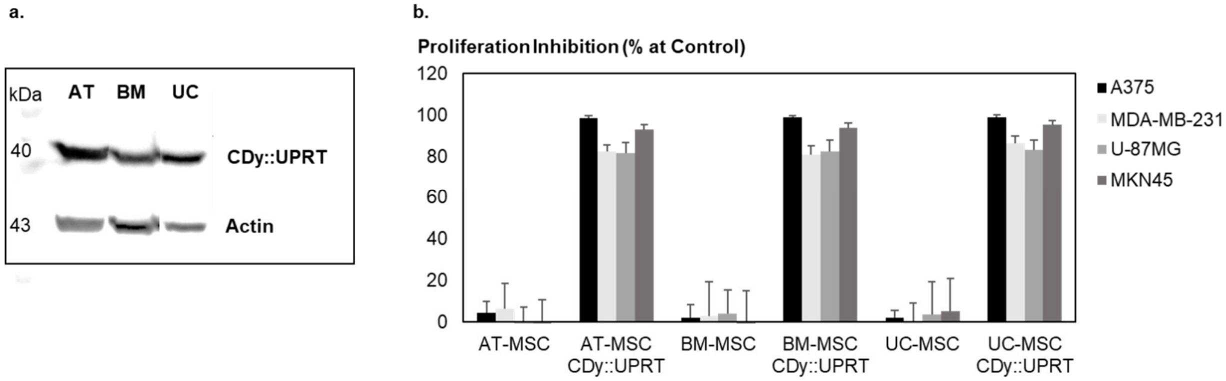

Figure S8. Comparable anticancer efficiency in stem cells from different sources. Adipose tissue (AT, Roosterbio), bone marrow (BM, Roosterbio), and UC (Umbilical cord, ATCC) derived MSCs were transfected in the presence of Enhancer. Twenty four hours post transfection, cells were trypsinised and collected for **(a)** western blot analysis. The cells were lysed for immunoblotting analysis with antibody targeting CDy and Actin. **(b)** In the same experiment, cells were harvested for coculture study with various cancer cell lines at the ratio of 1 MSC to 50 cancer cells. Cells were cocultured in the media containing 150 μg/mL of 5FC for 5 days. At the end of incubation, remaining cell number was evaluated spectrophotometrically by measuring the RFU of cells stained with Hoechst 33342 at wavelength Ex340/Em488. Conditions with un-transfected MSCs serve as control. Proliferation inhibition (%) was calculated according. Graph represents data collected from quadruplicates, mean ± SEM.

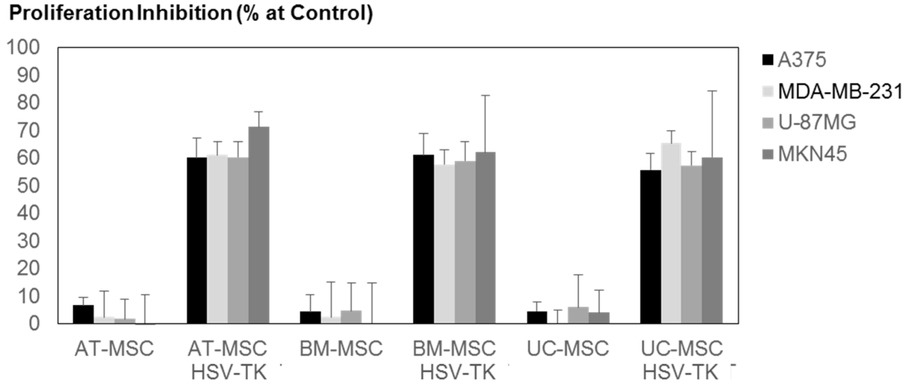

Figure S9. Comparable anticancer efficiency in various stem cells modified to express HSV-TK. AT-, BM- and UC-MSCs were transfected by pSELECT-zeo-HSV1tk (InvivoGen) in the presence of Enhancer. Twenty-four hours post transfection, MSCs were harvested and cocultured with various cancer cell lines at the ratio of 1 MSC to 50 cancer cells. Cells were

cocultured in the media containing 100 µg/mL of prodrug Ganciclovir (InvivoGen) for 5 days. At the end of incubation, remaining cell number based on RFU of cells stained with Hoechst 33342. Conditions with un-transfected MSC serve as controls. Data collected from biological quadruplicates are displayed as mean ± SEM.

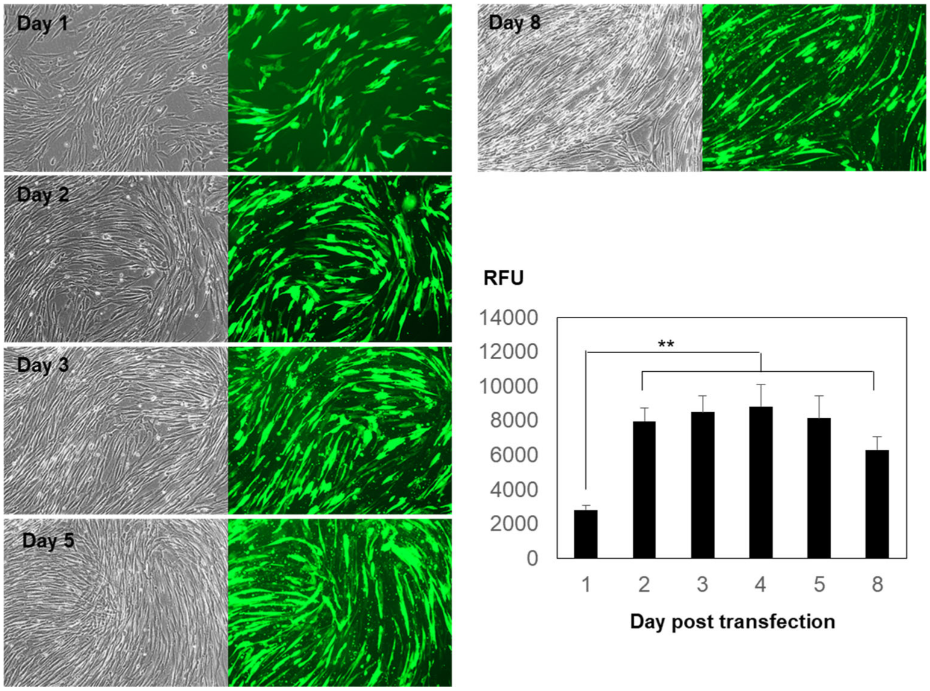

Figure S10. Prolonged expression CDy::UPRT::GFP in AT-MSCs. AT-MSCs were transfected with CDy::UPRT::GFP in the presence of Enhancer. On 1, 2, 3, 5, 8 day post transfection, the fluorescent and bright field images were captured. Relative Fluorescence Unit (RFU) of GFP expression was measured spectrophotometrically (Ex475/Em509) at nine areas of the culture. Graph represents mean of RFU ± SD for two biological replicates. Significant differences between the GFP expressions on various day post transfection were calculated using two tailed Student's t-test. **P < 0.01.

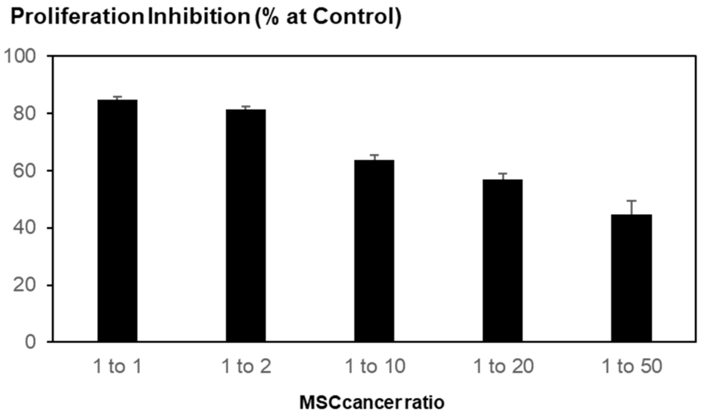

Figure S11. Growth inhibition of U251R by CDy::UPRT::GFP and 5FC. AT-MSCs were transfected with CD::UPRT::GFP in the presence of Enhancer. Twenty-four hours post transfection, cells were trypsinised and co-cultured with U251R (2500 cells/well) at the ratio of 1 MSC to 1, 2, 10, 20, 50 U251R cells. Cells were cocultured in the media containing 150 µg/mL of 5FC for 5 days. At the end of incubation, remaining cell number was evaluated spectrophotometrically by measuring the RFU of cells stained with Hoechst 33342 at wavelength Ex340/Em488. Conditions without 5FC treatment serve as control for each co-culture ratio. Proliferation inhibition (%) was calculated according. Graph represents data collected from quadruplicates, mean $\pm$ SD.

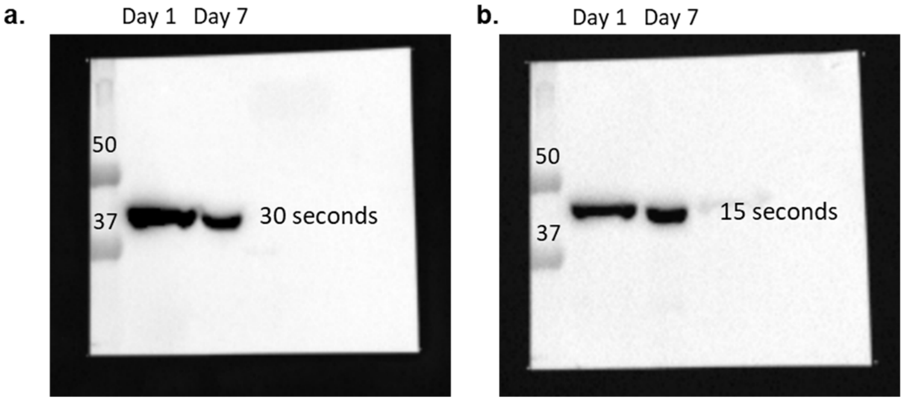

Figure S12. Full length blot for Figure 7a. (a) Whole cell lysates were collected and subjected to western blotting with antibody targeting CDy. After incubation with secondary antibody (conjugated to HRP), a mixture of peroxide solution and the luminol enhancer solution at 1:1 was added to the blot. The blot was exposed for 30 seconds and the image is displayed. After which, the primary and secondary antibodies were removed with stripping buffer. **(b)** The steps for western blotting were repeated with antibody targeting Actin. Blot was exposed for 15 seconds and image were captured.
